# Successful hepatitis B and C screening in the health check-up in the German primary care setting

**DOI:** 10.1016/j.jhepr.2024.101122

**Published:** 2024-05-24

**Authors:** Olaf Bätz, David Petroff, Katrin Jedrysiak, Ingmar Wolffram, Thomas Berg, Jan Kramer, Johannes Wiegand

**Affiliations:** 1 LADR Laboratory Group Dr. Kramer & Colleagues, Geesthacht, Germany; 2 Clinical Trial Centre Leipzig, University of Leipzig, Leipzig, Germany; 3 General Practitioner, Paderborn, Germany; 4 Division of Hepatology, Department of Medicine II, Leipzig University Medical Centre, Leipzig, Germany

**Keywords:** WHO, HBsAg, anti-HCV, HCV RNA, HBV DNA, elimination

## Abstract

**Background & Aims:**

A goal of the World Health Organization’s global hepatitis strategy is the elimination of chronic hepatitis C virus (HCV) infection by 2030. As part of its strategy, the Federal Joint Committee (Germany) decided to include hepatitis B and C screening in a preventive medical examination, which is performed at the primary care level in Germany. We investigated the results 1 year after implementation of screening between October 2021 and September 2022.

**Methods:**

HBsAg/HBV DNA and anti-HCV/HCV RNA screenings were identified by billing categories in 286,192 individuals of 11 ambulatory healthcare centers.

**Results:**

Compared to 30,106 HBsAg and 31,266 anti-HCV laboratory requisitions in the year 2018, the number of tests increased to 286,192 during the screening period. Compared to routine care, additional anti-HCV positive tests age dependently increased the tally by 98% (177 plus 170 positive cases in males) and 123% (96 plus 118 positive cases in females) in those aged 35-44 years up to 518% (17 plus 88 positive cases in males) and 514% (29 plus 149 positive cases in females) in those aged 75-84 years. Similar results were observed for HBsAg. Prevalences of HBsAg, anti-HCV and HCV RNA were 0.54%, 0.79% and 0.13%, respectively.

**Conclusions:**

A structured hepatitis screening program at the primary care level has been successfully established and leads to age- and-sex-dependent large additional effects compared to routine care.

**Impact and implications:**

Strategies to eliminate chronic hepatitis B and C virus infection are country specific and vary between clinical scenarios. Our analysis proves the efficacy of a screening program by primary care physicians compared to routine care in a low-prevalence country. This program should be accompanied by additional efforts in risk populations like people who inject drugs who are under-represented in the current screening approach.

## Highlights

• Screening efforts to diagnose unknown HBV and HCV infections in the general population are a major component of elimination efforts.
• A structured viral hepatitis screening program at the primary care level leads to large additional effects compared to routine care.
• Compared to 30,106 HBsAg and 31,266 anti-HCV lab requests in 2018, 286,192 tests were requested during the screening period.
• Prevalences of HBsAg, anti-HCV and HCV RNA were 0.54%, 0.79% and 0.13%, respectively, in the German population.

## Graphical abstract

**Figure:**
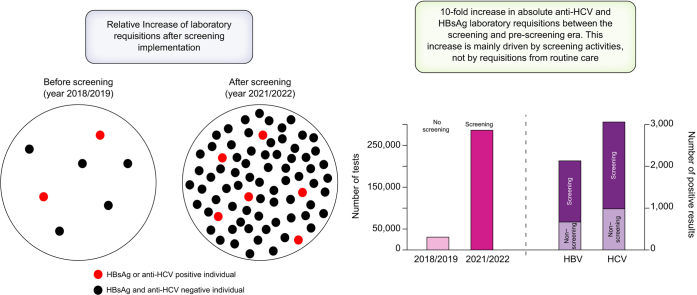

## Introduction

In 2016, the World Health Organization (WHO) published the global health sector strategy to eliminate chronic hepatitis C virus (HCV) infections as a public health threat by the year 2030.^1^ To support this initiative, the German Federal Ministry of Health devised the “BIS2030 strategy”, which includes screening strategies to diagnose so far unknown HCV infections.^2^

Screening is a major function of primary care in Germany, for example within the preventive medical examination “Gesundheitsuntersuchung”, formerly “Check-Up 35+”.^3^^,^^4^ It covers the patient’s medical history, an evaluation of risk factors, a physical examination, a cholesterol and blood glucose test, a spot urine test, and medical counselling about the results. In the year 2017/18, 50% of 24,966,730 eligible females and 44% of 21,349,871 males participated in the examination.^5^

In 2020, the Federal Joint Committee (Gemeinsamer Bundesausschuss, G-BA) introduced viral hepatitis screening within the “Check-Up 35+” as part of the national hepatitis elimination strategy and decided that statutory healthcare will reimburse a once in a lifetime screening for HBsAg and anti-HCV in patients 35 years or older.^2^^,^^6^ About 90% of the general population has statutory healthcare and can participate. If screening results are positive, PCR-based reflex testing of HBV DNA and HCV RNA is scheduled.

New billing categories were created to allow for implementation of the new screening strategy, which became effective in October 2021.^6^

The current project used these billing categories as a measure of the implementation of the viral hepatitis screening within the “Check-Up 35+” in the first year after its introduction.

## Patients and methods

Between 1^st^ October 2021 and 30^th^ September 2022 our project prospectively analyzed the database of 11 ambulatory healthcare centers of the LADR laboratory group, one of the five leading central laboratories in Germany which covers 7% of the German market.

Primary care physicians take a blood sample for HBsAg and anti-HCV screening within the “Check-Up 35+” if the patient opts in. These blood samples were identified by the billing code GOP 01865. If HBsAg and/or anti-HCV screening results are positive, the reflex test mentioned above can be identified by the codes GOP 01866 and 01867. This PCR reflex testing did not exist prior to the implementation of the “Check-Up 35+” screening program.

The once in a lifetime screening for hepatitis B and hepatitis C in the “Check-Up 35+” is associated with additional reimbursement for the primary care physician and can be performed irrespective of whether or not a patient is already known to be HBsAg or anti-HCV positive.

The results of the viral hepatitis screening were combined with demographic patient data (age and sex). Additional clinical data were not available.

In order to analyze how the number of “Check-Up 35+” related HBsAg and anti-HCV tests differ in the “clinical” (=“non-screening”) and screening scenarios, data with the screening GOP codes were compared to those without. We gathered data from three time spans:

a) Oct 2018 – Sept 2019 (before the COVID-19 pandemic)
b) Oct 2020 – Sept 2021 (during the COVID-19 pandemic)
c) Oct 2021 – Sept 2022 (implementation of HBV and HCV screening in the “Check-Up 35+”)

HBsAg and anti-HCV requisitions during the time periods 2018–2021 were based only on clinical indications without the possibility of screening in the “Check-Up 35+”, whereas those from 2021–2022 could be either.

In 2021, the study was approved by the Ethics Committee of the University of Leipzig (479/21-ek) and registered within the German Clinical Trial Register (DRKS, code 00029166).

### Endpoints

The primary endpoint was the proportion of HBsAg and anti-HCV screenings ordered within the study period compared to the total number of Check-Up lab requests.

The secondary endpoints were (a) the proportion of positive HBsAg and anti-HCV results, (b) the proportion of positive HBV DNA and HCV RNA results and (c) the above endpoints stratified according to age and sex.

### Sample size and statistics

We conservatively assumed that 150,000 check-up requests per year would be available, implying a width for the 95% CIs for the primary endpoint of less than 0.5 percentage points. This accuracy was considered adequate for our purposes. The choice of using data from an entire year ensured that there are no seasonal effects. With a prevalence for anti-HCV of 0.5%^7^ and assuming that two-thirds of the total screenings include hepatitis screenings, we expected about 500 positive anti-HCV results. The width for the 95% CIs on the estimate of positive HCV RNA cases was expected to be about 7 percentage points, since pilot data show that 20% of the 500 patients will have positive HCV RNA.

Estimates for proportions and their difference were based on observed frequencies and a Wilson score was used for construction of confidence intervals. The software R version 4.2.0 and the package “binom” were used for data analysis.^8^^,^^9^

## Results

A total of 286,192 individuals (56% female, mean age 61.2 ± 14.0 years) were screened for hepatitis B and C within the “Check-Up 35+”.

### Comparison between the “Check-Up 35+” time period and previous years

In the year 2018, 30,106 HBsAg and 31,266 anti-HCV laboratory requisitions were handled by the LADR laboratories. During the COVID-19 pandemic, there was a decline to 27,149 and 27,713. In comparison to these two observation periods, the HBsAg and anti-HCV requisitions in the “Check-Up 35+” screening both increased to n = 286,192.

To further illustrate the effect of the viral hepatitis screening within the “Check-Up 35+” program, we analyzed the number of positive HBsAg and anti-HCV results that occurred within routine care (“non-screening results”) and compared these to the “Check-Up 35+” numbers (“screening results”) (Table 1, Table 2).

**Table 1:** Number of HBsAg-positive patients from October 2021 until September 2022 by sex, age and “screening status”.

Age category (years)	Males			Females		
	Non-screening	Screening	Effect of screening: Increase of HBsAg-positive patients compared to the non-screening routine	Non-screening	Screening	Effect of screening: Increase of HBsAg-positive patients compared to the non-screening routine
35-44	108	169	156%	101	132	131%
45-54	106	205	193%	65	151	232%
55-64	83	202	243%	78	165	212%
65-74	47	142	302%	51	150	294%
75-84	14	43	307%	10	74	740%
>84	1	9	900%	4	21	525%

**Table 2:** Number of anti-HCV-positive patients from October 2021 until September 2022 by sex, age and “screening status”.

Age category (years)	Males			Females		
	Non-screening	Screening	Effect of screening: Increase of anti-HCV positive patients compared to the non-screening routine	Non-screening	Screening	Effect of screening: Increase of anti-HCV positive patients compared to the non-screening routine
35-44	174	170	98%	96	118	123%
45-54	209	254	122%	97	198	204%
55-64	134	313	234%	95	299	315%
65-74	55	176	320%	56	212	379%
75-84	17	88	518%	29	149	514%
>84	4	24	600%	17	75	441%

Compared to routine care alone, the number of anti-HCV positive tests increased by 98% (males) and 123% (females) in the age group 35-44 years up to 518% and 514% in the age group of 75-84 years (Table 2). Similar results were observed for HBsAg (Table 1).

### Hepatitis B screening results

The HBsAg and HBV DNA prevalence was 0.54% and 0.39%, while three HBV DNA samples could not be analyzed. Of patients with positive HBsAg, 72.7% were HBV DNA positive. The HBsAg prevalence according to sex was 0.64% in males and 0.46% in females. It was 0.48% (males) and 0.33% (females) for HBV DNA.

### Hepatitis C screening results

The anti-HCV and HCV RNA prevalence was 0.79% and 0.13%, while 11 HCV RNA samples could not be analyzed. Of patients with positive anti-HCV, 16.3% were HCV RNA positive. The anti-HCV prevalence according to sex was 0.91% in males and 0.69% in females. The corresponding values were 0.15% in males and 0.11% in females for HCV RNA.

## Discussion

Hepatitis B and C screening within the “Check-Up 35+” program is a successful component of the German “BIS2030” initiative to eliminate viral hepatitis. More than 280,000 HBsAg and anti-HCV screening requisitions in one of the five leading German laboratory networks within only 1 year indicate a high acceptance rate, and the strategy results in 10-times more requisitions compared to the years prior to implementation of the screening program. The screening effect is most evident if the increase of positive HBsAg and anti-HCV results is analyzed between routine care (“non-screening scenario”) and the screening within the “Check-Up 35+”. Reasons for success are a unified political will of healthcare authorities and statutory healthcare insurance, financial reimbursement, and the use of the established “Check-Up 35+” program at the primary care level. It is important that viral hepatitis screening was uniformly implemented throughout the whole country, because a decentralized healthcare system like in Italy with autonomy in planning, organizing, and financing healthcare services results in inconsistencies in how screening is implemented.^10^

The anti-HCV and – more importantly for treatment indication and viral eradication – the HCV RNA prevalence is above the threshold of >0.07%, above which a universal screening in any population is cost effective.^11^^,^^12^

Despite these encouraging results, several limitations remain: the screening results represent the number of positive tests, but they cannot differentiate between previously unknown cases and known infections that were simply re-tested. Nevertheless, we suppose that many patients are newly diagnosed, because the government’s central scientific institution for the safeguarding of public health, the Robert Koch Institute (RKI), observed an increase in hepatitis B and C infections since the implementation of the “Check-Up 35+” screening.^13^^,^^14^ The RKI discusses three major reasons for this observation: The “Check-Up 35+” initiative, immigration from the Ukraine, and an updated electronic surveillance system. Notably, an age-adjusted analysis shows that the increase is considerably greater in the age groups of 35–59 and 60–79 years compared to the age group of 20–34 years – thus in the categories in which our results indicate the greatest benefit of the screening program in comparison to routine care.

Diagnosis of HCV is made through primary care physicians, but therapy with direct-acting antivirals is typically initiated by specialists.^15^ Previous data indicate that newly diagnosed hepatitis C cases are often not successfully referred to secondary care.^16^^,^^17^ Therefore, primary care physicians should be encouraged to offer direct-acting antiviral therapy by themselves, considering that pangenotypic regimens have simplified HCV treatment.^15^^,^^18^ Similarly, hepatitis B-positive individuals are also not adequately linked to specialist care.^16^^,^^17^^,^^19^^,^^20^ HBV DNA assessment by PCR is often not performed in primary care.^20^ Knowledge about treatment indication, monitoring of treatment and support in the prescription of nucleos(t)ide analogues requires improvement.^21^

During the COVID-19 pandemic in the year 2021, only 28.6% of eligible patients participated in the “Check-Up 35+”. Prior to the pandemic in the year 2018, the participation rate was 34.3%.^5^ This rate must be improved by awareness initiatives.^10^^,^^15^ Knowledge about HCV infection and educational measures targeted towards primary care physicians are crucial to empower its elimination.^22^

The “Check-Up 35+” program addresses the general population, but not major viral hepatitis risk groups like people who inject drugs or men who have sex with men.^13^ These risk behaviors are under-reported, and individuals with lower socio-economic status are under-represented.^3^^,^^4^ Therefore, risk populations must be addressed by specifically designed approaches. Iceland and Australia can serve as role models for HCV treatment uptake in people who inject drugs,^23^^,^^24^ though recent data indicate high rates of reinfection.^25^

In conclusion, the hepatitis screening program at the primary care level has been successfully established and is a milestone towards eliminating viral hepatitis as a public threat in Germany. Additional targeted screening approaches in risk groups that are under-represented in primary care will be important to meet the goal of hepatitis B and C elimination.

## Abbreviations

Anti-HCV, anti-hepatitis C antibody; HBsAg, hepatitis B surface antigen; HBV, hepatitis B virus; HCV, hepatitis C virus.

## Financial support

The project was funded by an unrestricted research grant of Abbvie to JW (SA-003999).

## Conflict of interest

JW: Lecturer and advisory board member for Abbvie, Intercept/Advanz Pharma, GSK, Ipsen. TB: Receipt of grants/research supports: Abbvie, BMS, Gilead, MSD/Merck, Humedics, Intercept, Merz, Norgine, Novartis, Orphalan, Sequana Medical; Receipt of honoraria or consultation fees/advisory board: Abbvie, Alexion, Albireo, Bayer, Gilead, GSK, Eisai, Enyo Pharma, HepaRegeniX GmbH, Humedics, Intercept, Ipsen, Janssen, MSD/Merck, Novartis, Orphalan, Roche, Sequana Medical, SIRTEX, SOBI, and Shionogi; Participation in a company sponsored speaker’s bureau: Abbvie, Advance Pharma, Alexion, Albireo, Bayer, Gilead, Eisai, Falk Foundation, Intercept, Ipsen, Janssen, MedUpdate GmbH, MSD/Merck, Novartis, Orphalan, Sequana Medica, SIRTEX, and SOBI. Nothing to disclose: DP, OB, KJ, IW, JK.

Please refer to the accompanying ICMJE disclosure forms for further details.

## Authors’ contributions

Conceptualization: OB, DP, JK, JW. Methodology: OB, DP, JW. Data acquisition: OB, KJ, JK. Formal Analysis: DP, JW. Writing – Original Draft Preparation: DP, JW. Writing – Review & Editing: all authors.

## Data availability statement

Data can be shared upon individual request.
